# Emergency laparotomy clinical outcome according to patient characteristics, level of postoperative care and time of surgery

**DOI:** 10.1186/cc14626

**Published:** 2015-03-16

**Authors:** T Banerjee, M Templeton, C Gore

**Affiliations:** 1 St Mary´s Hospital, London, UK; 2 Imperial College Healthcare NHS Trust, London, UK

## Introduction

Emergency laparotomies have poor outcomes with variable postoperative critical care provision [1-3]. All patients requiring an emergency laparotomy with an estimated risk of death of >10% should go to critical care. Time of surgery should not affect standard of care [3,4]. In advance of the National Emergency Laparotomy Audit (NELA) results [2], our objective was to see whether the level of postoperative care and time of surgery affect outcome.

## Methods

Retrospective data were collected across the Imperial NHS trust for all emergency laparotomies over 3 months in 2014: length of stay in days (LOS); mortality; age; ASA; surgery time and postoperative care level, that is ward (L1), high dependency (L2), or ICU (L3). Statistical tests: Mann-Whitney, Pearson correlation (PCC) and multilinear regression analysis.

## Results

Seventy-one patients underwent surgery. Overall mortality was 13% and 70% of patients went to a L2/3 bed. More ASA 1/2 patients went to L1 and all ASA 4/5 went to L2/3. Median (IQR) for age was 61 (44 to 67) for L1, 65 (48 to 73) for L2/3 (*P *= 0.11), LOS was 10 (7 to 16) for L1, 19 (12 to 57) for L2/3 (*P *= 0.002), and mortality (%) was 0 for L1 and 18 for L2/3. For surgery between 08:00 and 17:59, 14 went to L1, 28 to L2/3. Mortality was 5% and LOS 15 (9 to 24). Between 18:00 and 21:59, two went to L1, 10 to L2/3. Mortality was 17% and LOS 22 (8 to 34). Between 22:00 and 07:59, five went to L1, 12 to L2/3. Mortality was 29% and LOS 15 (9 to 36). ASA strongly predicted mortality (*P *= 0.006, PCC 0.32). There was a negative correlation between postoperative destination and mortality with all deaths happening in those who went to L2/3 (*P *= 0.038 and PCC -0.25); however, sicker patients may have gone here. There was a strong correlation between mortality and time of surgery, night surgery being a strong predictor of mortality (PCC 0.31, *P *= 0.008). LOS can be predicted by a combination of ASA, age and care level (*P *= 0.027); the postoperative care regression coefficient was negative (-18.04, SE 10.41) with prolonged LOS in patients admitted to L2/3, which could also be explained by illness severity.

## Conclusion

Trust mortality is similar to that in the Emergency Laparotomy Network audit [1]. Higher ASA patients are appropriately going to L2/3 care. Baseline health status and time of surgery are the strongest predictors of mortality in emergency laparotomy patients.
